# Locoregional and systemic control after total neoadjuvant therapy with short-course radiotherapy for locally advanced rectal cancer: long-term outcomes from the LARCT-US study

**DOI:** 10.1093/bjs/znag014

**Published:** 2026-02-19

**Authors:** Israa Imam, Per J Nilsson, Tanweera Khan, Eva Angenete, Bengt Glimelius

**Affiliations:** Department of Immunology, Genetics and Pathology, Uppsala University, Uppsala, Sweden; Department of Oncology, Akademiska Sjukhuset, Uppsala, Sweden; Department of Pelvic Cancer, Division of Coloproctology, Karolinska University Hospital, Stockholm, Sweden; Department of Immunology, Genetics and Pathology, Uppsala University, Uppsala, Sweden; Department of Oncology, Akademiska Sjukhuset, Uppsala, Sweden; Department of Surgery, SSORG—Scandinavian Surgical Outcomes Research Group, Institute of Clinical Sciences, Sahlgrenska Academy, University of Gothenburg, Gothenburg, Sweden; Department of Surgery, Region Västra Götaland, Sahlgrenska University Hospital, Gothenburg, Sweden; Department of Immunology, Genetics and Pathology, Uppsala University, Uppsala, Sweden; Department of Oncology, Akademiska Sjukhuset, Uppsala, Sweden

## Abstract

**Background:**

Total neoadjuvant treatment (TNT) results in more complete responses and less risk of distant metastasis (DM) compared with chemoradiotherapy in locally advanced rectal cancer (LARC). The best schedule and the most suitable patients are unknown. In Sweden, after the closure of the RAPIDO trial, all hospitals in five out of six healthcare regions treated LARC patients with an abbreviated RAPIDO schedule, LARCT-US. Long-term data are reported.

**Methods:**

Between July 2016 and June 2020, LARC patients with at least one high-risk criterion for recurrence according to staging MRI (cT4, cN2, mesorectal fascia involvement <1 mm, extramural vascular involvement, lateral node involvement) received TNT consisting of 5 × 5 Gy followed by four cycles of CAPOX/six cycles of FOLFOX.

**Results:**

Curatively treated patients (437 of 462) were analysed after a median of 6.5 (interquartile range 5.9–7.2) years of follow-up. cT4 was seen in 53.5%. Sixty-two patients with a cCR entered a watch-and-wait programme (21 patients with regrowth) and 375 patients underwent primary surgery. At 5 years, of the 437 patients, locoregional recurrence (LRR) occurred in 26 patients (5.9% (95% c.i. 3.7% to 8.2%)) and DM occurred in 108 patients (24.7% (95% c.i. 20.7% to 28.7%)). The distal resection margin was ≤10 mm in 8.3% of patients after a sphincter-saving procedure (a lower percentage than in RAPIDO). The 109 patients (24.9%) with a complete response (48 patients with a cCR sustained for >1 year after the start of radiotherapy and 61 patients with a pCR) had excellent outcomes (0% with LRR and 3.7% with DM).

**Conclusion:**

TNT consisting of 5 × 5 Gy followed by four cycles of CAPOX/six cycles of FOLFOX resulted in excellent locoregional and distant control, despite inclusion of more advanced tumours than previous TNT studies. The low LRR risk in LARCT-US could be explained by more adequate distal resection margins practiced at Swedish centres.

## Teaser

This study evaluates long-term outcomes of the LARCT-US protocol, an abbreviated RAPIDO schedule, in locally advanced rectal cancer patients. After a median of 6.5 years of follow-up, locoregional recurrence was low (5.9%), compared with the TNT arm of RAPIDO (10.8%). This may be explained by more adequate distal resection margins practiced at Swedish centres.

## Introduction

In locally advanced rectal cancer (LARC), the initial treatment has traditionally been chemoradiotherapy (CRT). This neoadjuvant approach improves the resectability of non-resectable tumours, decreases local recurrence rates, and improves long-term outcomes. It may also induce a cCR, with the potential for non-operative management (organ preservation or ‘watch and wait’ (W&W))^1,2^. The reference treatment (CRT consisting of about 50 Gy with concomitant fluoropyrimidine), often accompanied by adjuvant chemotherapy, has been challenged by total neoadjuvant treatment (TNT). A few randomized trials have compared CRT (±adjuvant chemotherapy) and TNT^3–6^. However, different inclusion criteria have been used, due to an absence of consensus on what constitutes LARC, and different TNT schedules have been used^7^. Therefore, the most appropriate patients and tumours for TNT are unknown.

Some TNT trials have used short-course radiotherapy (scRT; 5 × 5 Gy in 1 week)^3–5^ followed by oxaliplatin-containing chemotherapy (CAPOX/FOLFOX), while others have used similar chemotherapy, or a triple combination (chiefly FOLFIRINOX), before or after CRT^6^. Collectively, the trials show that complete response (both pCR and cCR) rates increase and distant metastasis (DM) rates decrease^2^. Locoregional recurrence (LRR) is generally not influenced, although the RAPIDO trial reported increased LRR rates (from 6% in the CRT arm to 11% in the TNT arm) after 5 years of follow-up^8^. Some trials have reported improved disease-free survival or disease-related treatment failure, while overall survival (OS) has not clearly been improved^4–6,9^.

In the RAPIDO trial, 920 patients with at least one risk criterion according to MRI (cT4, cN2, mesorectal fascia involvement <1 mm (MRF+), extramural vascular involvement (EMVI+), lateral node involvement (LN+)) were randomized between TNT (scRT + 6 cycles of CAPOX/9 cycles of FOLFOX) and CRT (±adjuvant chemotherapy). After the inclusion phase was completed in June 2016, all Swedish centres from five out of six healthcare regions began treating LARC patients fulfilling the inclusion criteria for RAPIDO with an abbreviated schedule (scRT + 4 cycles of CAPOX/6 cycles of FOLFOX, rather than scRT + 6 cycles of CAPOX/9 cycles of FOLFOX) until the release of the results of the RAPIDO trial (June 2020). Reasons for the abbreviation of the chemotherapy were that several colon cancer trials found that 3 months of adjuvant oxaliplatin/fluorouracil was non-inferior to 6 months of treatment (the International Duration Evaluation of Adjuvant Therapy (IDEA) consortium^10^) and a fear that tumour progression could happen for poorly responding tumours during prolonged futile chemotherapy^11^. The hypothesis tested in LARCTUS was that this abbreviation would provide similar results as the TNT arm in RAPIDO. Patients were treated either according to a phase II protocol (locally advanced rectal cancer treatment—Uppsala style (LARCT-US)) with written patient consent or according to the same protocol in routine care (ad modum LARCT-US (AdmL))^11^. The aim of this planned 5-year update of the large, prospective, and population-based LARCT-US/AdmL cohort was to investigate potential causes for the increased LRR risk reported in RAPIDO and to better understand the most appropriate patients and tumours to treat with TNT. The hypothesis was that no increased risk of LRR would be seen in LARCT-US/AdmL.

## Methods

### Patients, treatment, and immediate outcome

During the 4-year interval, 273 patients fulfilling the RAPIDO characteristics in five out of six Swedish healthcare regions were prospectively included in LARCT-US (clinicaltrials.gov NCT03729687) and 189 similarly treated patients (AdmL) were identified from the Swedish Colorectal Cancer Registry (SCRCR). Inclusion and exclusion criteria have been published previously^11^. Briefly, patients aged ≥18 years with an Eastern Cooperative Oncology Group (ECOG) performance status of 0 or 1, with biopsy-proven primary rectal cancer (≤15 cm from the anal verge), and with at least one high-risk criterion for recurrence according to staging MRI (cT4, cN2, MRF+, EMVI+, LN+) were eligible. Treatment consisted of external radiotherapy of 5 × 5 Gy during week 1 followed by four cycles of CAPOX (oxaliplatin 130 mg/m^2^ on day 1 and capecitabine 1000 mg/m^2^ twice daily for 14 days). Alternatively, six cycles of modified FOLFOX-6 (oxaliplatin 85 mg/m^2^, leucovorin 200 mg/m^2^, and bolus 5-fluorouracil 400 mg/m^2^ on day 1 followed by fluorouracil 2400 mg/m^2^ for 46 h on days 1–3) could be administered. Tumour response was evaluated 1–2 weeks after the conclusion of chemotherapy.

**Fig. 1:**
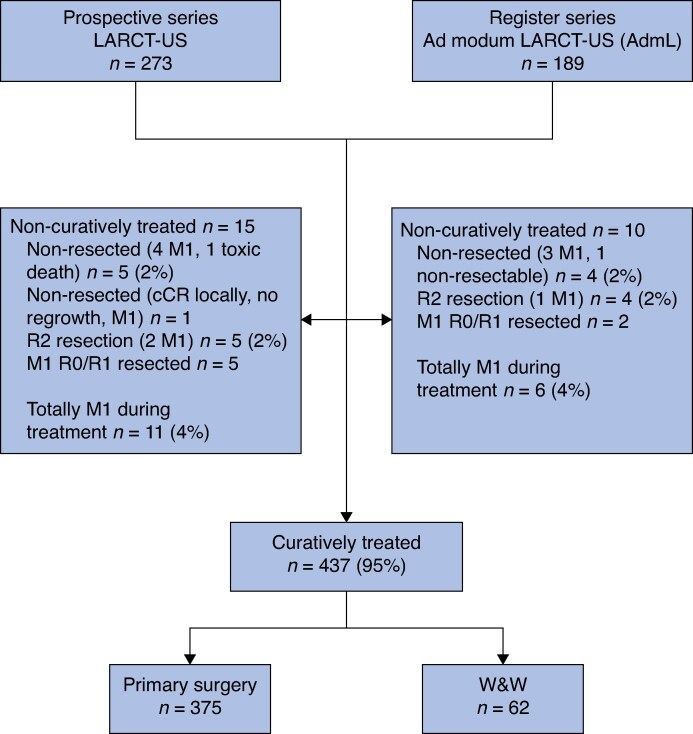
Flow chart

Treatment compliance and immediate outcome have been reported^11^. The primary outcome variable was the complete response rate (pCR or cCR sustained for >1 year after the start of radiotherapy). Twenty-five patients were considered to be non-curatively treated because of appearance of DM, non-resection, or R2 resection, leaving 437 potentially curatively treated patients available for long-term follow-up (*Fig. 1*). Of these 437 patients, 375 underwent primary surgery (R0/R1) and 62 entered a W&W programme. As the baseline characteristics and outcomes were similar between LARCT-US and AdmL patients^11^, the two cohorts were analysed together.

### Follow-up and registration in the SCRCR

Patients were routinely followed according to the Swedish national care programme for colorectal cancer, meaning thoracoabdominal CT at 1 year and 3 years with carcinoembryonic antigen (CEA) measurements, and a clinical follow-up without mandatory radiology after 5 years. Colonoscopy was performed at 3 years and thereafter depending on findings. Additional radiology and/or other investigations were performed according to clinical suspicion. For patients with a cCR who entered the W&W programme, the Swedish guidelines stipulated follow-up using MRI, sigmoidoscopy, CEA measurements, and digital rectal examination every 3 months for 2 years and thereafter biannually up to 5 years. In addition to registration in the SCRCR, cCR patients were registered in the Swedish Watch-and-Wait Registry (NCT03125343)^12^. For LARCT-US patients, follow-up information was registered in case report forms (CRFs); the SCRCR was used as a data source for AdmL patients and was also used as a supplementary data source for LARCT-US patients.

### Statistical methods

The outcomes included risk of and time to DM and LRR (time to recurrence (TTR)), OS, location of any recurrence, and OS after DM or LRR. Time to event was measured from the date of surgery for the patients who underwent primary surgery and from the start of preoperative radiotherapy for the patients who entered the W&W programme. Patients were censored at the date of last follow-up (September 2025) if no event had occurred. The reverse Kaplan–Meier method was used to calculate the median follow-up time. The median survival time was calculated using the Kaplan–Meier method.

Univariable Cox regression analyses were used to investigate the associations of clinicopathological factors with mortality and recurrence for all patients and separately for the patients who were operated on (all and only non-pCR) and the results are presented in forest plots, with HRs and corresponding 95% confidence intervals. Cox regression was used for univariable analyses, except when subgroups had no or very few events; Firth’s penalized Cox regression was then applied to obtain more reliable estimates^13^.

Variables with a *P* value <0.100 in univariable analyses were considered for inclusion in the multivariable models; however, if there was a risk of reduced model interpretability, that is a risk of creating unstable or difficult-to-interpret models, variables with few events, collinearity, or redundant covariates were excluded. Variables considered most relevant clinically were prioritized. The confidence intervals for binomial proportions were calculated using either the exact Clopper–Pearson method for small samples (<30) or when event counts were low (≤2), as it ensures nominal coverage even in such constrained scenarios, or the Wilson score method for larger sample sizes with more events. A *P* value <0.050 was considered statistically significant. All analyses were performed using R version 4.4.1.

### Ethical approval

The research ethics committee at Uppsala University approved the LARCT-US protocol (Dnr 2016/305). All patients provided informed consent before treatment. In an amendment, retrospective collection of data from the SCRCR for all similarly treated patients was approved (Dnr 2016/305/9). This included the retrieval of information that was missing in the SCRCR from treating hospitals. The study was registered at clincialtrials.gov (NCT03729687). Reporting is in accordance with STROBE guidelines.

## Results

Of the 437 curatively treated patients in LARCT-US/AdmL (*Fig. 1*), 375 patients underwent primary surgery (R0 92.3%, R1 7.7%) and 62 patients entered a W&W programme (*Table 1*). Resections performed included 184 anterior resections (49.1%), 156 abdominoperineal excisions (41.6%), and 35 Hartmann’s procedures (9.3%). Extended surgery, that is beyond total mesorectal excision (TME) and/or simultaneous *en bloc* resection of other organs, was performed in 102 patients (27.2%).

**Table 1:** Baseline characteristics of 437 LARC patients who underwent primary surgery (R0/R1, M0; *n* = 375) or entered the W&W programme (*n* = 62) after TNT according to the LARCT-US concept

	All patients	Primary surgery (R0/R1)	W&W
Total, *n*	437	375	62
**Age (years)**			
≤69	309 (70.7)	260 (69.3)	49 (79)
≥70	128 (29.3)	115 (30.7)	13 (21)
**Sex**			
Male	256 (58.6)	222 (59.2)	34 (55)
Female	181 (41.4)	153 (40.8)	28 (45)
ECOG performance status*			
0	190 (73.6)	159 (72.6)	31 (79)
1	66 (25.6)	58 (26.5)	8 (21)
Not known	2 (0.8)	2 (0.9)	0 (0)
**ASA grade**			
I	58 (13.2)	57 (15.2)	1 (2)
II	235 (53.8)	226 (60.3)	9 (15)
≥III	77 (17.6)	74 (19.7)	3 (5)
Not known	67 (15.3)	18 (4.8)	49 (79)
**Clinical T and N status**			
cT3 N0	13 (3.0)	10 (2.7)	3 (5)
cT2–3 N+	186 (42.6)	149 (39.7)	37 (60)
cT4 N0	28 (6.4)	21 (5.6)	7 (11)
cT4 N+	210 (48.1)	195 (52.0)	15 (24)
**Risk factors**			
cT4	238 (54.5)	216 (57.6)	22 (35)
cN2	257 (58.8)	236 (62.9)	21 (34)
MRF+	335 (76.7)	288 (76.8)	47 (76)
EMVI+	231 (52.9)	204 (54.4)	27 (44)
LN+	96 (22.0)	85 (22.7)	11 (18)
**Number of risk factors**			
1	88 (20.1)	60 (16.0)	28 (45)
2	121 (27.7)	108 (28.8)	13 (21)
3	110 (25.2)	98 (26.1)	12 (19)
4	91 (20.8)	84 (22.4)	7 (11)
5	27 (6.2)	25 (6.7)	2 (3)
**Tumour level**			
Low (0–4 cm)	129 (29.5)	108 (28.8)	21 (34)
Mid (5–9 cm)	159 (36.4)	134 (35.7)	25 (40)
High (10–15 cm)	149 (34.1)	133 (35.5)	16 (26)
**Tumour length**			
≤40 mm	99 (22.7)	69 (18.4)	31 (50)
41–69 mm	203 (46.5)	180 (48.0)	23 (36)
≥70 mm	90 (20.6)	88 (23.5)	1 (2)
Not known	45 (10.3)	38 (10.1)	7 (11)
CEA*			
≤3.6 µg/l	115 (44.6)	87 (39.7)	28 (72)
3.7–5.0 µg/l	37 (14.3)	32 (14.6)	5 (13)
>5.0 µg/l	98 (38.0)	93 (42.5)	5 (13)
Not known	8 (3.1)	7 (3.2)	1 (3)
**Response**			
Sustained cCR†	48 (11.0)	–	48 (79)
pCR	61 (14.0)	61 (16.3)	–
Non-complete response	328 (75.1)	314 (83.7)	14 (21)
**NAR score‡**			
Low	150 (34.3)	99 (26.4)	51 (84)
Intermediate	163 (37.3)	154 (41.1)	9 (13)
High	124 (28.4)	122 (32.5)	2 (3)
Compliance (≥75%)*			
Yes	228 (88.4)	191 (87.2)	37 (95)
No	30 (11.6)	28 (12.8)	2 (5)

Values are *n* (%) unless otherwise indicated. R0 corresponds to a resection margin of >1 mm and R1 corresponds to a resection margin of ≤1 mm. *Only recorded in LARCT-US (*n* = 258) of which 219 patients underwent planned resection surgery and 39 patients entered the W&W programme. †cCR sustained for >1 year after the start of radiotherapy; if of shorter duration, the patient was included in the non-complete response group. ‡Patients who entered the W&W programme and achieved a cCR were considered to have a low NAR score. LARC, locally advanced rectal cancer; W&W, watch and wait; TNT, total neoadjuvant treatment; ECOG, Eastern Cooperative Oncology Group; MRF+, mesorectal fascia involvement <1 mm; EMVI+, extramural vascular involvement; LN+, lateral node involvement; CEA, carcinoembryonic antigen; NAR, neoadjuvant rectal.

### Recurrences

**Fig. 2:**
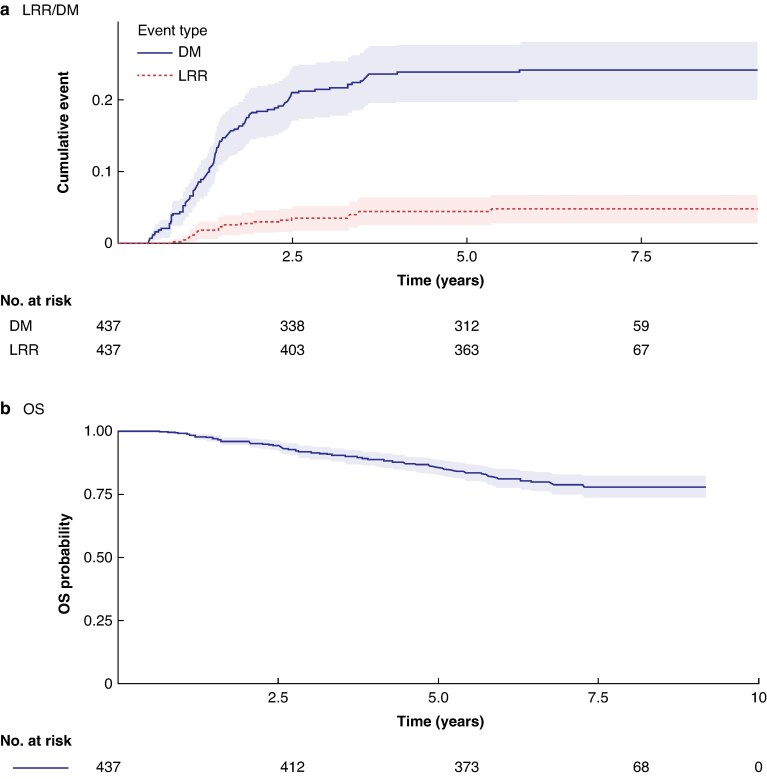
Risk of recurrence and survival **a** Risk of LRR and DM. **b** OS. Time to event was measured from the date of surgery for the patients who underwent primary surgery and from the start of preoperative radiotherapy for the patients who entered the W&W programme. LRR, locoregional recurrence; DM, distant metastasis; OS, overall survival; W&W, watch and wait.

In September 2025, at a median follow-up of 6.5 (interquartile range (i.q.r.) 5.9–7.2, range 5.1–9.2) years, 117 patients (26.8%) had a recurrence. Of 437 patients, 26 patients (5.9% (95% c.i. 3.7% to 8.2%)) had LRR and 108 patients (24.7% (95% c.i. 20.7% to 28.7%)) had DM (*Table 2*). The median TTR was 1.4 (i.q.r. 1.0–2.1, range 0.4–5.8) years from the start of radiotherapy (*Fig. 2a*). Of the 26 patients who had LRR, 17 (65%) also had DM (before LRR in 5 patients, synchronously with LRR (within 30 days) in 9 patients, and metachronously with LRR in 3 patients).

**Table 2:** LRR and DM recurrence and LRR and DM recurrence risks for clinical factors of 437 LARC patients who received TNT according to the LARCT-US concept

	Total	LRR	DM
Recurrence	117 (26.8)	26 (5.9)	108 (24.7)
**Age (years)**			
≤69	80 (25.9)	15 (4.9)	75 (24.3)
70+	37 (28.9)	11 (8.9)	33 (25.8)
**Sex**			
Male	65 (25.4)	13 (5.1)	62 (24.2)
Female	52 (28.7)	13 (7.2)	46 (25.4)
ECOG performance status*			
0	52 (27.3)	7 (3.7)	49 (25.8)
1	19 (29)	9 (14)	17 (26)
**ASA grade**			
I	20 (34)	4 (7)	18 (31)
II	60 (25.5)	14 (6.0)	56 (23.8)
≥III	29 (38)	6 (8)	26 (34)
Not known	8 (12)	2 (3)	8 (12)
**Clinical T and N status**			
cT3 N0	2 (15)	0 (0)	2 (15)
cT2–3 N+	45 (24.2)	10 (5.4)	39 (21.0)
cT4 N0	5 (18)	1 (4)	5 (18)
cT4 N+	65 (31.0)	15 (7.1)	62 (29.5)
**Risk factors**			
cT4	70 (29.4)	16 (6.7)	67 (28.2)
cN2	74 (28.8)	17 (6.6)	69 (26.8)
MRF+	89 (26.6)	17 (5.1)	86 (25.7)
EMVI+	73 (31.6)	14 (6.1)	69 (29.9)
LN+	30 (31)	9 (9)	28 (29)
**Number of risk factors**			
1	19 (22)	7 (8)	14 (16)
2	28 (23.1)	5 (4.1)	26 (21.5)
3	31 (28.2)	5 (4.5)	30 (27.3)
4	28 (31)	5 (5)	27 (30)
5	11 (41)	4 (15)	11 (41)
**Tumour level**			
Low (0–4 cm)	38 (29.5)	13 (10.1)	35 (27.1)
Mid (5–9 cm)	34 (21.4)	5 (3.1)	32 (20.1)
High (10–15 cm)	45 (30.2)	7 (4.7)	41 (27.5)
**Tumour length**			
≤40 mm	30 (30.0)	6 (6.0)	29 (29.0)
41–69 mm	56 (27.6)	12 (5.9)	49 (24.1)
≥70 mm	23 (26)	7 (8)	22 (25)
Not known	8 (18)	1 (2)	8 (18)
CEA*			
≤3.6 µg/l	24 (20.9)	5 (4.3)	23 (20.0)
3.7–5.0 µg/l	11 (30)	4 (11)	9 (24)
>5.0 µg/l	35 (36)	6 (6)	34 (35)
Not known	1 (13)	1 (13)	0 (0)
Compliance (≥75%)*			
Yes	57 (25.0)	10 (4.4)	54 (23.7)
No	14 (47)	6 (20)	12 (40)

Values are *n* (%). *Only recorded in LARCT-US. LRR, locoregional recurrence; DM, distant metastasis; LARC, locally advanced rectal cancer; TNT, total neoadjuvant treatment; ECOG, Eastern Cooperative Oncology Group; MRF+, mesorectal fascia involvement <1 mm; EMVI+, extramural vascular involvement; LN+, lateral node involvement; CEA, carcinoembryonic antigen.

Of the 375 patients who underwent primary surgery, 109 patients (29.1%) had a recurrence (*Table 3*): LRR (26 patients (7%), occurring at a median of 1.2 (i.q.r. 1.0–2.2, range 0.6–5.3) years) and/or DM (100 patients (26.7%); occurring at a median of 1.3 (i.q.r. 1.0–1.9, range 0.4–5.8) years). Among the 184 patients who underwent an anterior resection, 10 patients (5.4%) had LRR. Only 1 of 35 patients (3%) who underwent a Hartmann’s procedure had LRR (*Table 3*). For patients who were operated on with a pCR, 0% of patients had LRR, compared with 7.9% of patients who were operated on without a pCR; DM rates were also considerably lower when a pCR was achieved (2% (1 patient) *versus* 31.7%).

**Table 3:** LRR and DM recurrence risks for surgical factors and treatment response of 437 LARC patients who underwent primary surgery (R0/R1, M0; *n* = 375)) or entered the W&W programme (*n* = 62) after TNT according to the LARCT-US concept

	Total	LRR	DM
Total	117 (26.8)	26 (5.9)	108 (24.7)
**Treatment**			
Entered the W&W programme	8 (13)	0 (0)	8 (13)
Planned resection surgery	109 (29.1)	26 (6.9)	100 (26.7)
Type of resection*			
Anterior resection	58 (31.5)	10 (5.4)	54 (29.3)
Abdominoperineal excision	45 (28.8)	15 (9.6)	41 (26.3)
Hartmann’s procedure	6 (17)	1 (3)	5 (14)
Surgery beyond TME*			
No	73 (27.4)	14 (5.3)	66 (24.8)
Yes	34 (33.3)	11 (10.8)	32 (31.4)
Not known	2 (29)	1 (14)	2 (29)
Resection plane*			
Mesorectal	49 (24.5)	7 (3.5)	46 (23.0)
Intramesorectal	13 (50)	3 (12)	13 (50)
Muscular	7 (39)	4 (22)	6 (33)
Not known	40 (30.5)	12 (9.2)	35 (26.7)
Distal resection margin*†			
≤10 mm	5 (31)	1 (6)	5 (31)
>10 mm	51 (28.6)	7 (4.0)	47 (26.9)
Not known	8 (29)	3 (11)	8 (29)
Residual tumour classification*			
R0 (>1 mm)	96 (27.7)	22 (6.4)	87 (25.1)
R1 (≤1 mm)	13 (45)	4 (14)	13 (45)
Tumour deposits*			
No	66 (21.5)	17 (5.5)	60 (19.5)
Yes	43 (66)	9 (14)	40 (62)
Not known	0 (0)	0 (0)	0 (0)
Pathological EMVI+*			
No	51 (20.7)	13 (5.3)	46 (18.7)
Yes	42 (49)	8 (9)	40 (47)
Not known	16 (36)	5 (11)	14 (32)
Pathological T stage*			
ypT0	1 (2)	0 (0)	1 (2)
ypT1–2	14 (18)	4 (5)	12 (15)
ypT3	73 (39.2)	16 (8.6)	69 (37.1)
ypT4	21 (46)	6 (13)	18 (39)
Pathological N stage*			
ypN0	36 (14.8)	10 (4.1)	31 (12.8)
ypN1	50 (49.5)	12 (11.9)	47 (46.5)
ypN2	23 (74)	4 (13)	22 (71)
**Response**			
Sustained cCR‡	3 (6)	0 (0)	3 (6)
pCR	1 (2)	0 (0)	1 (2)
Non-complete response	113 (34.5)	26 (7.9)	104 (31.7)
**NAR score§**			
Low	8 (5.3)	1 (0.7)	8 (5.3)
Intermediate	38 (23.3)	10 (6.1)	33 (20.2)
High	71 (57.2)	15 (12.1)	67 (54.0)

Values are *n* (%). *Only patients who underwent planned resection surgery. †Patients who underwent either an anterior resection or a Hartmann’s procedure (*n* = 219); distal resection margin as reported by the pathologist. ‡cCR sustained for >1 year after the start of radiotherapy; if of shorter duration, the patient was included in the non-complete response group. §Patients who entered the W&W programme and achieved a cCR were considered to have a low NAR score. LRR, locoregional recurrence; DM, distant metastasis; LARC, locally advanced rectal cancer; W&W, watch and wait; TNT, total neoadjuvant treatment; TME, total mesorectal excision; EMVI+, extramural vascular involvement; NAR, neoadjuvant rectal.

Of the 62 patients who entered the W&W programme, local regrowth (not considered as LRR if an R0 resection could be performed) was detected within 1 year in 14 patients (67% of all regrowth) and later in 7 patients. In all patients with regrowth after initial W&W, R0 surgery was performed and no subsequent LRR occurred. Overall, DM occurred in eight W&W patients (13%) (*Table 3*), occurring at a median of 1.8 (i.q.r. 1.2–2.1) years after the start of radiotherapy. Six instances of DM were associated with regrowth, of which four were within the first year.

### Survival

The 5-year OS was 85.4% (95% c.i. 82.1% to 88.7%) (*Fig. 2b*). Patients with a complete response had a significantly better OS compared with patients with a non-complete response (*Fig. S1*). As details of treatments for either LRR or DM were not recorded in the CRF and SCRCR data on post-recurrence treatment were limited, only OS could be analysed. The median OS was 3.0 (i.q.r. 1.8–4.5) years after DM and 2.0 (i.q.r. 0.6–3.4) years after LRR.

### First recurrence and localization of DM and LRR

Ninety-six patients had DM as first recurrence, 12 patients had LRR as first recurrence, and 9 patients had both DM and LRR as first recurrence (*Fig. S2a*). Most instances of DM were pulmonary, followed by hepatic, peritoneal, and extra-regional lymph nodal (*Fig. S2b*). The location of LRR was available for 15 of 26 patients. Details are presented in *Fig. S2c*.

### Associations between pretreatment and post-treatment characteristics and the risks of LRR and DM

The response to treatment (complete response) had the strongest association with recurrence (*Table 3* and *Fig. S3*). Some individual risk criteria, the total number of risk criteria, pretreatment CEA, and treatment compliance were also associated with recurrence (*Tables 2*, *3* and *Fig. S3*). In the multivariable analyses, reaching a complete response was associated with DM and LRR. A tumour level <5 cm was marginally associated with LRR (*Table S1*).

For patients who underwent primary surgery, more advanced ypT and ypN, as well as the presence of tumour deposits, were associated with an increased risk of recurrence, as was a high neoadjuvant rectal (NAR) score. A pCR was associated with a very low risk, with no patients with LRR and only one patient (2%) with DM (in this patient, the quality of the pathological examination was poor). A suboptimal resection specimen, that is with an intramesorectal or a muscular resection plane, and an R1 resection were associated with increased risks of both LRR and DM, while the type of resection was not (*Table 3* and *Figs S4*, *S5*). Surgery beyond TME had a limited influence on recurrence risks (*Table 3*). In multivariable analyses, tumour deposits and tumour regression grades 0 and 1 were associated with DM, while a suboptimal resection plane was associated with both DM and LRR (*Table S2*).

As the recurrence risks were minimal in patients with a complete response (cCR/pCR), the associations with recurrence risks and some pathological variables were also explored in patients with a non-complete response. Tumour deposits were statistically significantly associated with DM in these patients (*Table 3* and *Fig. S6*). Tumour regression grade 0 was associated with fewer recurrences and was statistically significant in the multivariable analyses for DM, while tumour deposits were statistically significant for both DM and LRR (*Table S3*).

The distal resection margin (as reported by the pathologist) for the 219 patients operated on with sphincter-preserving surgery (anterior resection or Hartmann’s procedure) was ≤10 mm for 16 patients (8.3% of 191 with known distance). The LRR and DM risks were 6% (95% c.i. 0.2% to 30.2%) and 31% (95% c.i. 11.0% to 58.7%) respectively if ≤10 mm and 4.0% (95% c.i. 2.0% to 8.0%) and 26.9% (95% c.i. 20.8% to 33.9%) respectively if >10 mm (*Table 3*). If the distance was unknown, the rates were similar (data not shown).

## Discussion

In this almost nationwide cohort study of patients with LARC at high risk of recurrence treated with an abbreviated neoadjuvant schedule (scRT + 4 cycles of CAPOX/6 cycles of FOLFOX), excellent outcomes were observed, including a high complete response rate^11^, a low risk of LRR, and a low risk of DM. The low LRR risk, despite more advanced tumours, contrasts with the unexpectedly high rate of 10.8% after TNT in the RAPIDO trial that included an additional two cycles of CAPOX/three cycles of FOLFOX. This apparently high rate in RAPIDO, in contrast to 5.8% after CRT, has been the subject of detailed investigation. It appears that a too narrow surgical distal resection margin after sphincter-preserving surgery at a few hospitals in the Netherlands^14^ might be the main contributor and plausible explanation. The observation in the RAPIDO study that abdominoperineal excision was performed in 45.7% of Sweden patients compared with only 29.6% of Dutch patients supports this explanation. In the LARCT-US/AdmL cohort the abdominoperineal excision rate was similar to that among Swedish RAPIDO patients and few narrow distal resection margins were observed. Thus, surgeons should bear in mind that, despite an apparent significant tumour shrinkage after TNT, it is possible that non-detectable viable tumour cells may remain distal to the tumour visible using MRI and a strategy change from abdominoperineal excision to anterior resection following a good, albeit not complete, response to TNT may be associated with an increased risk of LRR.

A fragmented tumour response to preoperative treatment is frequently seen^15^. What constitutes a safe distal resection margin in patients not responding completely is not known, although a 1 cm rule has been recommended, but based upon limited data^16,17^. Whether the type of preoperative treatment influences what constitutes a safe distal resection margin is not known and should be the focus of future investigation.

The distribution of sites of systemic recurrence in this cohort was similar to that in RAPIDO^18^. Thus, in the TNT arm in RAPIDO, and in this study, most instances of DM were in the lungs, whereas the liver as the initial site was more common after CRT in RAPIDO^18^. The reasons for this are not known. OS after recurrence, for both LRR and DM, was slightly longer in this study than in RAPIDO^8,18^. Speculatively, this may reflect improvements in curatively intended and palliative treatments over time after recurrence.

A rationale for abbreviating the number of chemotherapy cycles was that progression may occur for poorly responding tumours during the prolonged treatment, resulting in an increased risk of non-radical surgery and subsequent LRR^11^. In LARCT-US/AdmL, four cycles of CAPOX provided the same chance of a complete response as six cycles in RAPIDO. Whether four cycles are as efficient as six cycles in eradicating subclinical disease is not known^2^. Consequently, mid-treatment MRI is recommended and, if no regression is seen after four cycles, the benefit of the additional cycles is questionable and surgery should be performed.

Which patients benefit most from intense TNT compared with CRT, or scRT and a delay, is also not known. The aim of the neoadjuvant treatment is of importance. If the aim is organ preservation, small tumour size and low CEA are the greatest determinants^11,19,20^. Even if non-operative management is also preferred by patients with LARC, the major aim is to prevent DM more efficiently compared with adjuvant chemotherapy, as demonstrated in two randomized trials (RAPIDO and UNICANCER-PRODIGE 23)^4,6,9,21^. In these studies, a more advanced tumour stage/presence of risk factors was associated with DM, but the associations were not strong^9,18^. This report shows similar associations with DM. The presence of adverse pathological signs (such as ypT4, ypN2, EMVI+, and tumour deposits), a high NAR score, and a suboptimal resection plane confer a recurrence risk of around 50% or higher. Currently, this high risk of DM could indicate closer surveillance after surgery, but additional treatments to reduce the risk are lacking. The relevance of pathological variables after pretreatments other than TNT for recurrence risk has been reported previously^22,23^. The recent data on LRR and the association with distal resection margin reported from the RAPIDO trial^14^ together with the LRR rate from this cohort indicate that TNT with the aim of increasing the rate of sphincter-preserving surgery is questionable.

The prospective study design of LARCT-US and the register-based data of AdmL introduce some limitations, particularly for AdmL for which the SCRCR was the main data source. However, despite this, the cohort in the present study probably reflects real life better than randomized trial cohorts. Furthermore, the thorough registration of outcome data makes comparisons with the RAPIDO trial possible.

The results of LARCT-US/AdmL for the entire LARC population in Sweden, several of whom were truly advanced, sometimes requiring irradiation to large volumes and extended surgery, show that this treatment is not well tolerated by all patients in routine care. Compliance with the abbreviated schedule used in the Swedish LARCT-US/AdmL real-world cohort was inferior to that reported from the RAPIDO trial^11^. This observation indicates that the intense chemotherapy schedules included in randomized trials may not be universally applicable on a population level. Finally, these data and those of the RAPIDO trial suggest that baseline information regarding the original tumour should be considered when the surgical approach after TNT is determined for non-completely responding tumours.

## Supplementary Material

znag014_Supplementary_Data

## Data Availability

Data are available upon reasonable request.
