# Supplementary material for: Locoregional and systemic control after total neoadjuvant therapy with short-course radiotherapy for locally advanced rectal cancer: long-term outcomes from the LARCT-US study
Source: Br J Surg. 2026 Feb 19;113(3):znag014. doi: 10.1093/bjs/znag014 (PMC13017942; doi:10.1093/bjs/znag014)
Supplement: znag014_Supplementary_Data [file znag014_supplementary_data.docx]

**Locoregional and systemic control following total neoadjuvant therapy with short-course radiotherapy for locally advanced rectal cancer – long-term outcomes from the LARCT-US study**

Israa Imam, M.D., PhD^1,2^, Per J. Nilsson, M.D., PhD^3^, Tanweera Khan, M.D., PhD^1,2^, Eva Angenete, M.D., PhD^4,5^, Bengt Glimelius, M.D., PhD^1,2^

^1^Department of Immunology, Genetics and Pathology, Uppsala University, Uppsala, Sweden

^2^Department of Oncology, Akademiska Sjukhuset, Uppsala, Sweden

^3^Department of Pelvic Cancer, Division Coloproctology, Karolinska University Hospital, Stockholm, Sweden

^4^Department of Surgery, SSORG - Scandinavian Surgical Outcomes Research Group, Institute of Clinical Sciences, Sahlgrenska Academy, University of Gothenburg, Gothenburg, Sweden

^5^Region Västra Götaland, Sahlgrenska University Hospital, Department of Surgery, Gothenburg, Sweden

**Corresponding author.** Israa Imam, israa.imam@igp.uu.se. **ORCID ID** **0000-0002-0232-2391.**

**Supplementary Materials - Index**

| **Supplementary Figures and Tables** |  |
| --- | --- |
| Figure S1 | *page 2* |
| Figure S2 | *page 3* |
| Figure S3 | *page 4* |
| Table S1 | *page 5* |
| Figure S4 | *page 5* |
| Figure S5 | *page 6* |
| Table S2 | *page 7* |
| Figure S6 | *page 7* |
| Table S3 | *page 8* |

**Supplementary Figures and Tables**

**
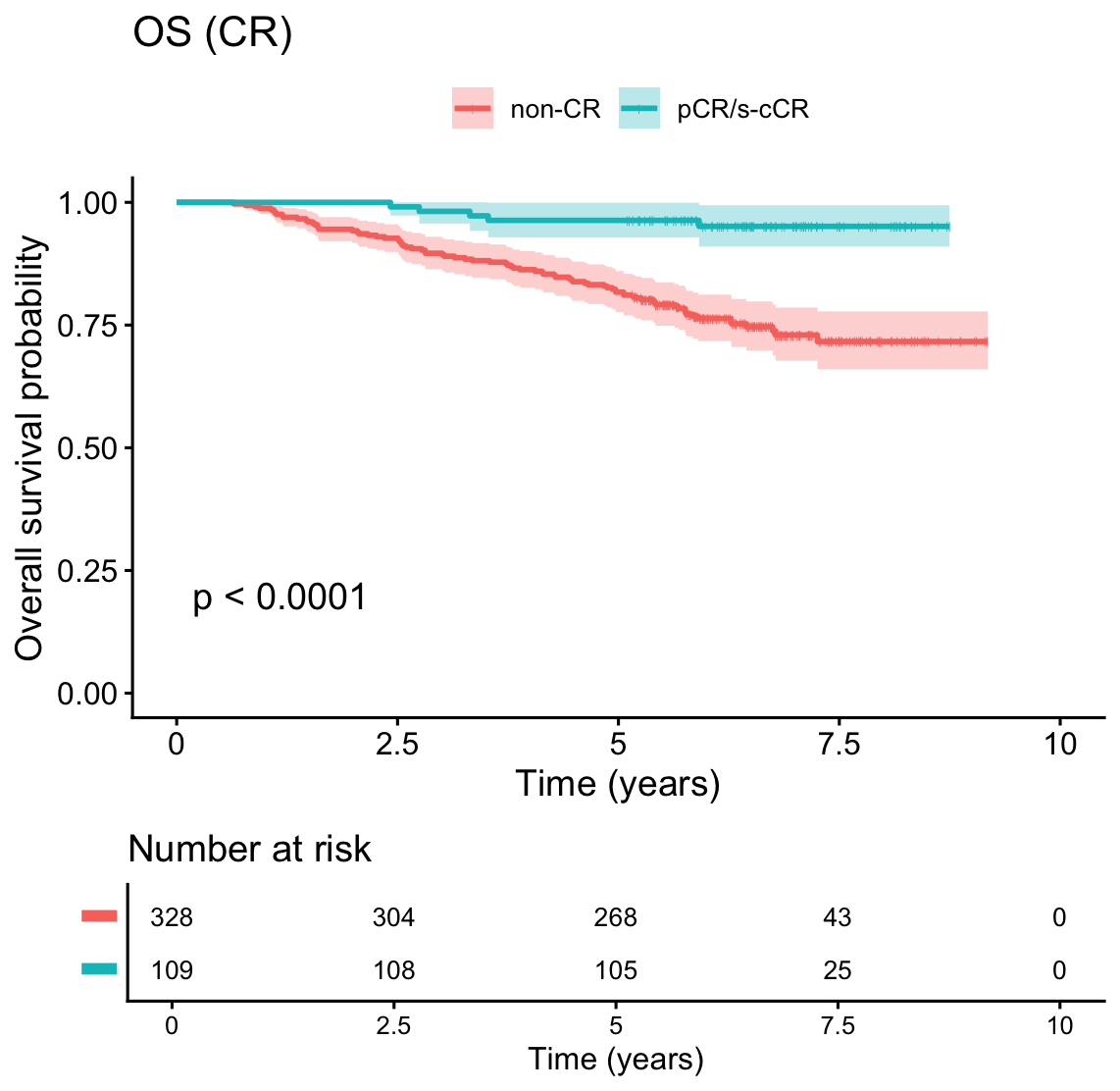
**

**Figure S1. Overall survival (OS) for patients with non-complete remission (non-CR) or complete remission (CR); pathological CR (pCR) or sustained clinical CR (s-cCR) with a duration exceeding 1 year from the start of radiotherapy. Time to event was considered from the date of surgery for primarily operated patients or from start of preoperative radiotherapy for patients entering the watch-and-wait program.**


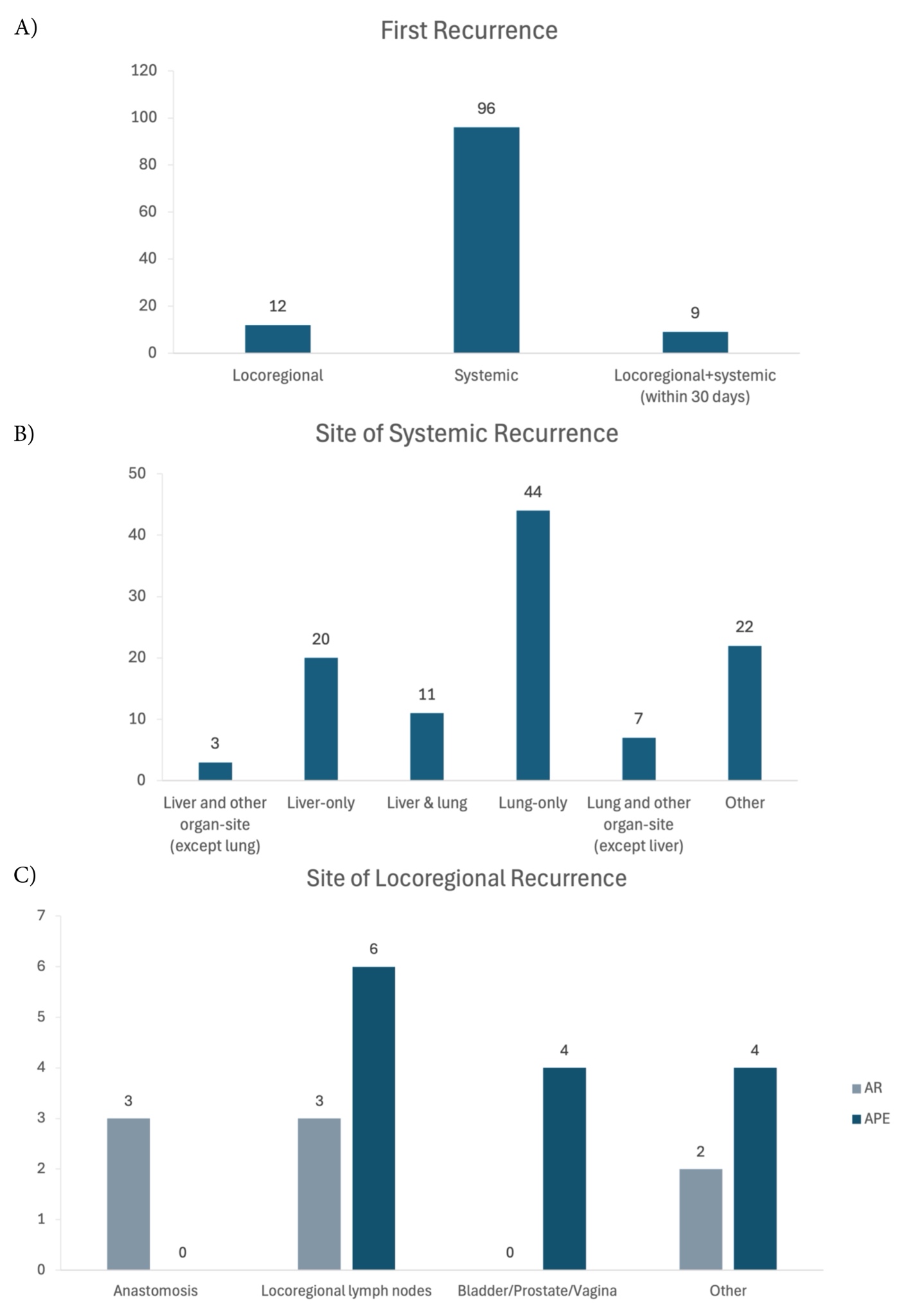


**Figure S2. A) Type of first recurrence, B) site of systemic recurrence and C) site of locoregional recurrence (only LARCT-US).** Anterior resection. AR; Abdominoperineal excision, APE. Of the 16 LARCT-US patients that had a locoregional recurrence, 7 had an AR and 9 had an APE (information on location of locoregional recurrence missing for one patient that had an AR).

**
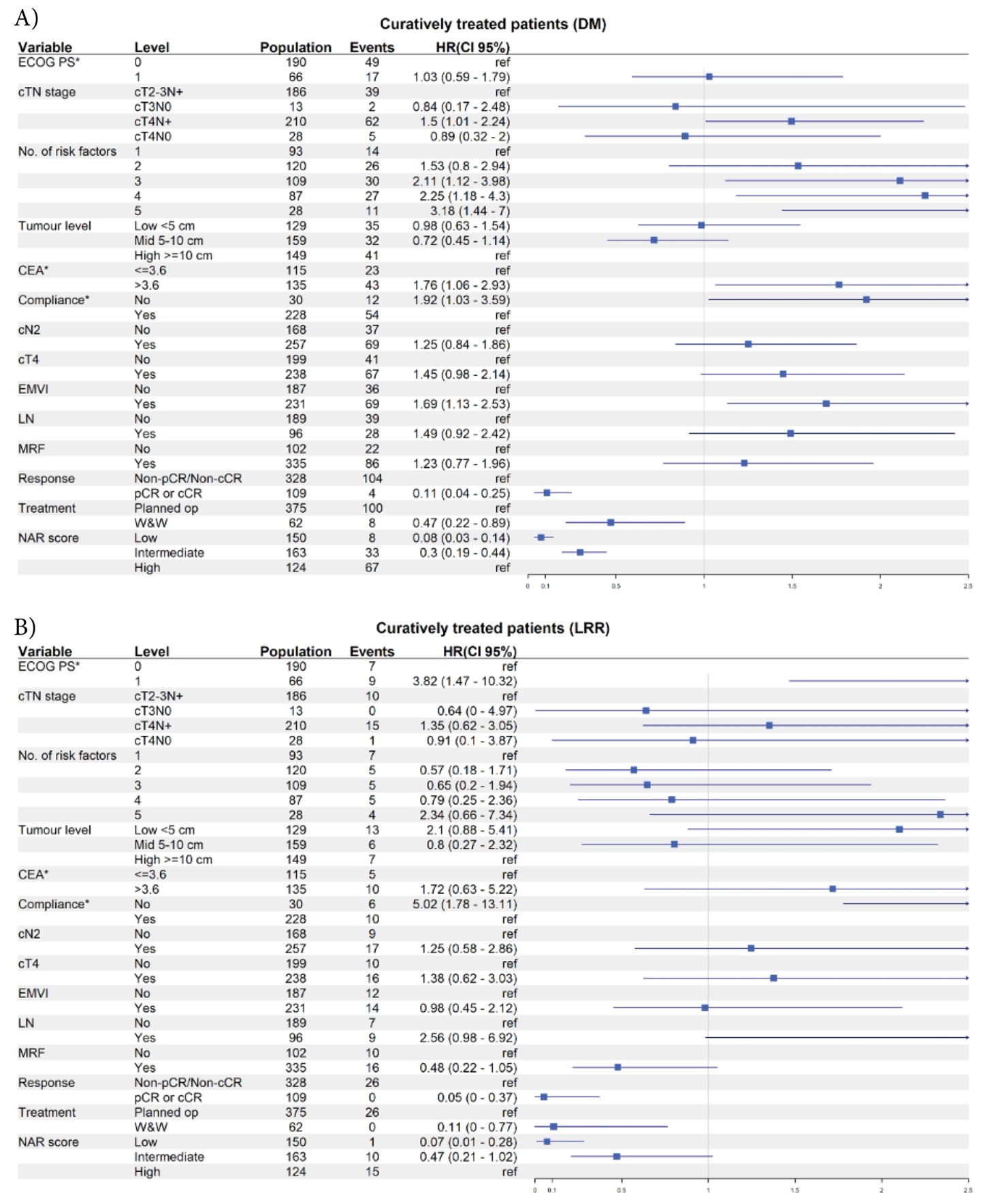
**

**Figure S3. Forest plot of univariable Cox regression in 437 curatively treated patients for A) distant metastasis (DM) and for B) locoregional recurrence (LRR).**

*Subgroup analysis (only LARCT-US)

**Table S1. Multivariable Cox regression in 437 curatively treated patients for distant metastasis (DM) and for locoregional recurrence (LRR).**

| Term | HR | CI low | CI high | *p*-value |
| --- | --- | --- | --- | --- |
| DM |  |  |  |  |
| cTN-stage cT3N0 | 0.991 | 0.198 | 3.131 | 0.989 |
| cTN-stage cT4N0 | 0.821 | 0.297 | 1.851 | 0.658 |
| cTN-stage cT4N+ | 1.008 | 0.627 | 1.633 | 0.975 |
| Number of risk factors 2 | 1.199 | 0.623 | 2.406 | 0.593 |
| Number of risk factors 3 | 1.785 | 0.910 | 3.656 | 0.093 |
| Number risk factors 4 | 1.565 | 0.752 | 3.373 | 0.234 |
| Number risk factors 5 | 2.203 | 0.887 | 5.420 | 0.088 |
| Response pCR or cCR | 0.115 | 0.038 | 0.265 | **<0.001** |
| LRR |  |  |  |  |
| Tumour level Low <5 cm | 2.400 | 1.006 | 6.185 | **0.048** |
| Tumour level Mid 5-10 cm | 0.881 | 0.297 | 2.547 | 0.813 |
| Response pCR or cCR | 0.049 | <0.001 | 0.349 | **<0.001** |

Variables with a *P*-value <0.10 for DM and LRR in the univariable Cox regression were included.

**
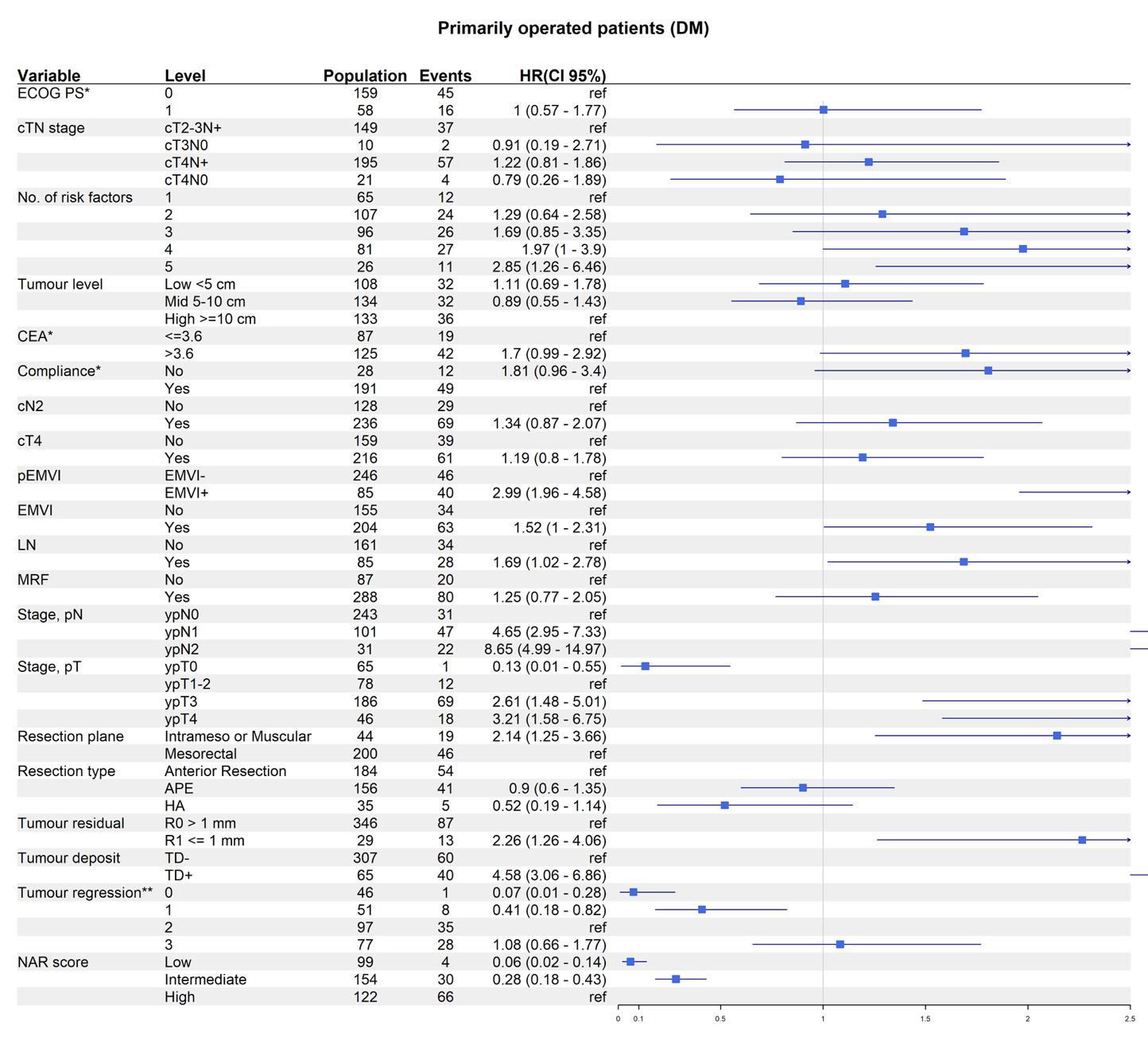
**

**Figure S4. Forest plot of univariable Cox regression in 375 primarily operated patients (W&W patients were excluded even if they later had regrowth and had subsequent surgery) for distant metastasis (DM).**

*Subgroup analysis (only LARCT-US)

**Tumour regression according to AJCC.

**
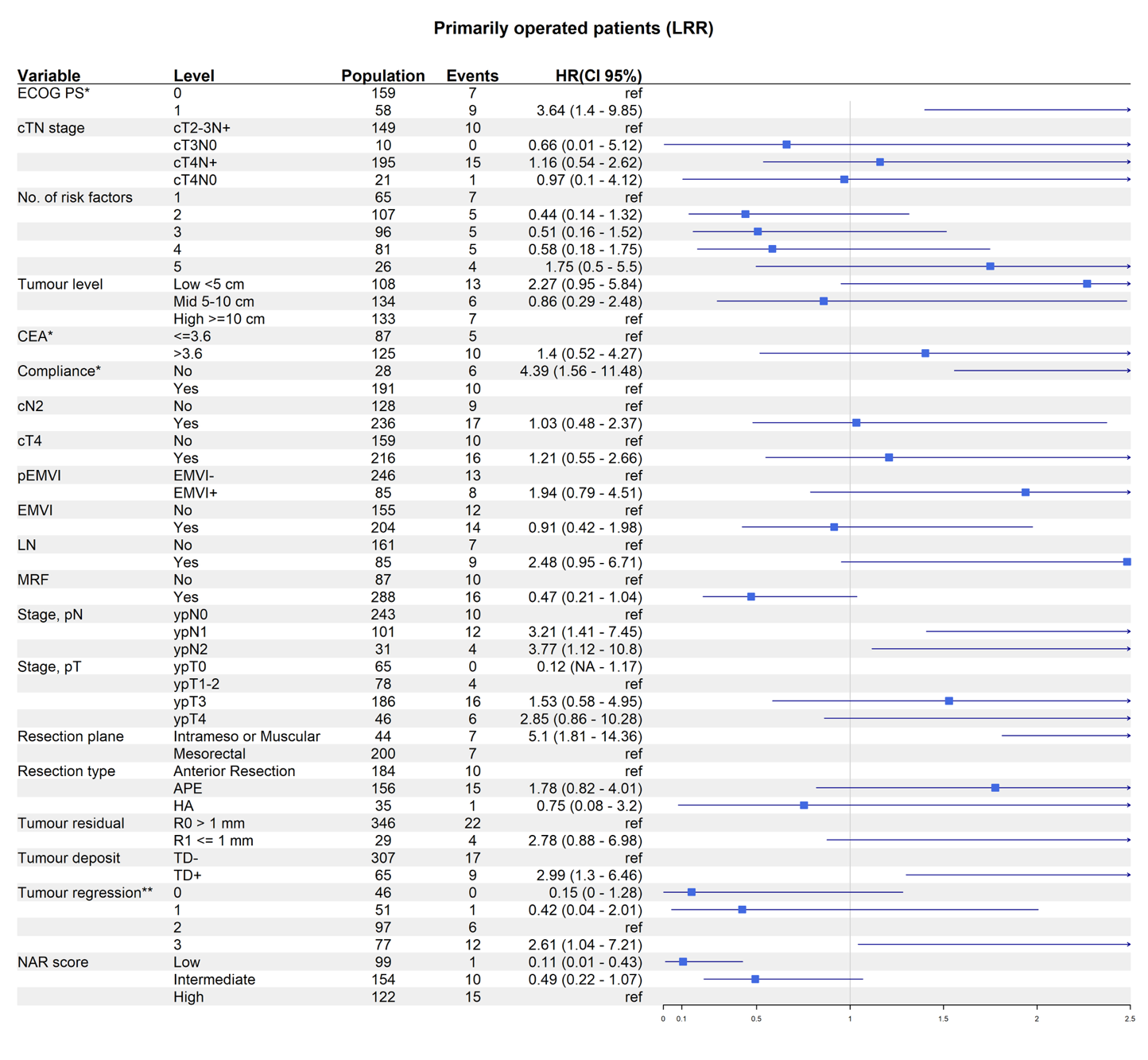
**

**Figure S5. Forest plot of univariable Cox regression in 375 primarily operated patients (W&W patients were excluded even if they later had regrowth and had subsequent surgery) for locoregional recurrence (LRR).**

*Subgroup analysis (only LARCT-US)

******Tumour regression according to AJCC.

**Table S2. Multivariable Cox regression in 375 primarily operated patients for distant metastasis (DM) and for locoregional recurrence (LRR).**

| Term | HR | CI low | CI high | *p*-value |
| --- | --- | --- | --- | --- |
| DM |  |  |  |  |
| Number of risk factors 2 | 1.064 | 0.364 | 3.398 | 0.912 |
| Number of risk factors 3 | 0.919 | 0.291 | 3.141 | 0.888 |
| Number of risk factors 4 | 0.924 | 0.278 | 3.288 | 0.898 |
| Number of risk factors 5 | 1.893 | 0.461 | 7.854 | 0.371 |
| Tumor length 41–69 mm | 0.696 | 0.311 | 1.601 | 0.385 |
| Tumor length 70+ mm | 0.746 | 0.281 | 1.955 | 0.549 |
| Resection plane Intramesorectal or Muscular | 3.292 | 1.414 | 7.299 | **0.007** |
| Residual tumour R1 ≤ 1 mm | 1.463 | 0.598 | 3.292 | 0.388 |
| Tumour deposit TD+ | 5.528 | 2.423 | 12.642 | **<0.001** |
| Tumour regression* 0 | 0.189 | 0.020 | 0.810 | **0.022** |
| Tumour regression* 1 | 0.251 | 0.049 | 0.838 | **0.022** |
| Tumour regression* 3 | 1.467 | 0.676 | 3.129 | 0.328 |
| pEMVI+ | 1.157 | 0.576 | 2.285 | 0.678 |
| LRR |  |  |  |  |
| Resection plane Intramesorectal or Muscular | 4.873 | 1.569 | 14.72 | **0.007** |
| Tumour deposit TD+ | 3.067 | 0.862 | 9.385 | 0.080 |
| Residual tumour R1 ≤ 1 mm | 3.170 | 0.914 | 9.470 | 0.067 |

Variables with a *P*-value <0.10 for DM and P-value <0.05 for LRR in the univariable Cox regression were included.

*****Tumour regression according to AJCC.

**
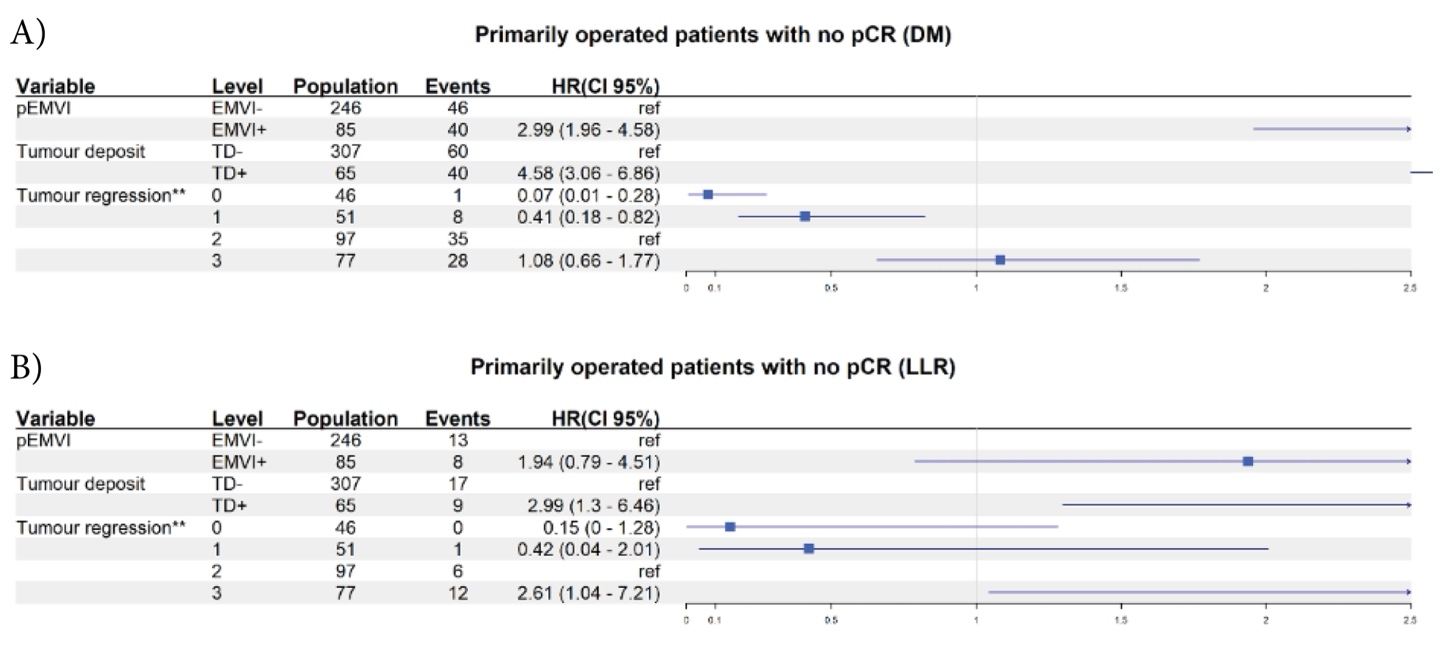
**

**Figure S6. Forest plot of univariable Cox regression in 314 primarily operated patients with non-pCR for A) distant metastasis (DM) and B) for locoregional recurrence (LRR).**

**Tumour regression according to AJCC.

**Table S3. Multivariable Cox regression in 314 primarily operated patients with no pCR for distant metastasis (DM) and for locoregional recurrence (LRR).**

| Term | HR | CI low | CI high | *p*-value |
| --- | --- | --- | --- | --- |
| DM |  |  |  |  |
| Tumour deposit TD+ | 4.647 | 2.656 | 8.077 | **<0.001** |
| Tumour regression* 0 | 0.140 | 0.015 | 0.551 | **0.002** |
| Tumour regression* 1 | 0.516 | 0.213 | 1.099 | 0.089 |
| Tumour regression* 3 | 1.553 | 0.889 | 2.694 | 0.121 |
| EMVI+ | 1.018 | 0.584 | 1.753 | 0.948 |
| LRR |  |  |  |  |
| Tumour deposit TD+ | 2.780 | 1.194 | 6.080 | **0.019** |
| Residual tumour R1 ≤ 1 mm | 2.278 | 0.709 | 5.842 | 0.151 |

Variables with a *P*-value <0.10 for DM and P-value <0.05 for LRR in the univariable Cox regression were included.

*****Tumour regression according to AJCC.
